# Inulin alters gut microbiota to alleviate post‐stroke depressive‐like behavior associated with the IGF‐1‐mediated MAPK signaling pathway

**DOI:** 10.1002/brb3.3387

**Published:** 2024-01-17

**Authors:** Rong Shao, Xiongchang Tan, Minfu Pan, Jiawen Huang, Liu Huang, Binyu Bi, Xiaohua Huang, Jie Wang, Xuebin Li

**Affiliations:** ^1^ School of Clinical Medicine Youjiang Medical University for Nationalities Baise Guangxi China; ^2^ Department of Neurology The Affiliated Hospital of Youjiang Medical University for Nationalities Baise Guangxi China; ^3^ Biological Molecule Laboratory Guangxi University Key Laboratory of High Incidence Prevention and Control Research in Western Guangxi Baise Guangxi China

**Keywords:** brain‐derived neurofactors, gut microbiota, IGF‐1, inulin, MAPK pathway, post‐stroke depression

## Abstract

**Introduction:**

Gut microbiota dysbiosis is a key factor of the pathogenesis of post‐stroke depression (PSD). PSD is associated with increased hippocampal neuronal apoptosis and decreased synaptic connectivity. Inulin can be involved in hippocampal neuron protection through the microbiome–gut–brain axis. However, the neuroprotective effects of inulin in PSD are still to be further investigated.

**Methods:**

By utilizing the GEO public database, we identify differentially expressed genes in the hippocampus following inulin intake. This can help us discover key signaling pathways through functional enrichment analysis. Furthermore, we validate the expression levels of signaling molecules in a rat model of PSD and examine the effects of inulin on behavioral changes and body weight. Additionally, conducting a microbiome analysis to identify significantly different microbial populations and perform correlation analysis.

**Results:**

The intake of inulin significantly up‐regulated mitogen‐activated protein kinase signaling pathway in the hippocampus. Inulin changed in the gut microbiota structure, leading to an increase in the abundance of *Lactobacillus* and Clostridium_sensu_stricto_1 in the intestines of PSD rats, while decreasing the abundance of *Ruminococcus* UCG_005, Prevotella_9, Oscillospiraceae, and Clostridia UCG_014. Furthermore, the inulin diet elevated levels of insulin‐like growth factor 1 in the serum, which showed a positive correlation with the abundance of *Lactobacillus*. Notably, the consumption of inulin‐enriched diet increased activity levels and preference for sugar water in PSD rats, while also reducing body weight.

**Conclusion:**

These findings highlight the potential therapeutic benefits of inulin in the management of depression and emphasize the importance of maintaining a healthy gut microbiota for PSD.

## Graphical abstract

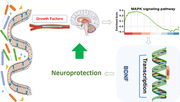

## 1 INTRODUCTION

**FIGURE 1:**
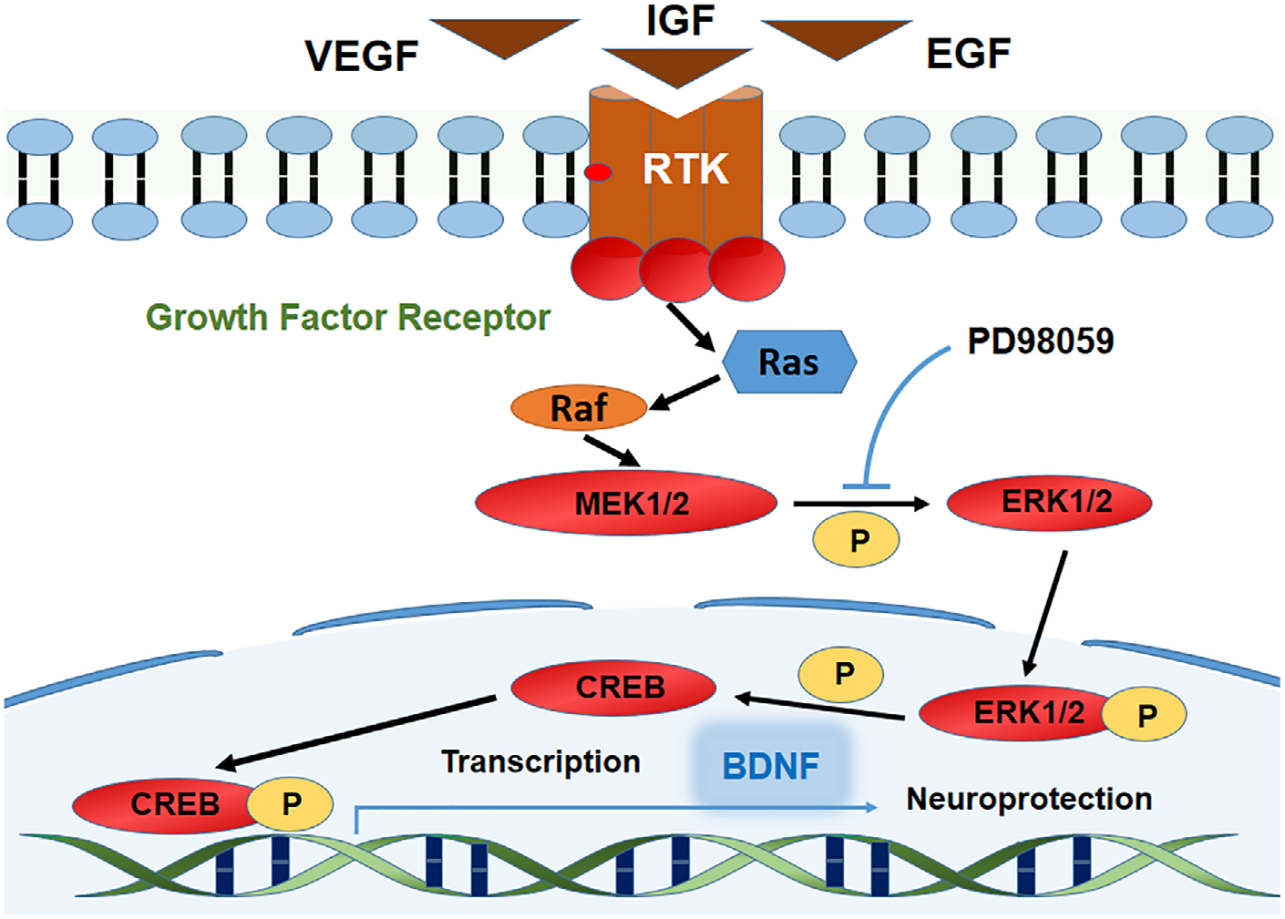
Mitogen‐activated protein kinase (MAPK) signaling pathway: The MAPK signaling pathway involves the binding of various growth factors to growth factor receptors (tyrosine kinase receptors), forming complexes that can activate Ras‐dependent synergistic effects. The Ras‐dependent cascade reaction is the Ras–Raf–MEK–ERK signal transduction pathway. In this process, phosphorylated ERK1/2 (P‐ERK1/2) can further phosphorylate cAMP response element‐binding (CREB) and promote the transcription of brain‐derived neurotrophic factor (BDNF) mRNA. The activation reaction is represented by a black arrow, whereas PD98059 is indicated by a blue line representing its inhibitory effect on this process.

Post‐stroke depression (PSD) is a mental disorder that occurs after a stroke, characterized by reduced interest, decreased motor activity, low mood, and impaired cognitive function. PSD hindered the recovery process after stroke and increased the risk of disability (Ahn et al., 2015; Woranush et al., 2021). Statistics showed that over 30% of stroke survivors suffer from PSD (Guo et al., 2022; Zhang et al., 2013). The pathogenesis of PSD is complex, involving psychological factors, neuroendocrine dysfunction, inflammatory responses, as well as alterations in neurotransmitters and neuroplasticity in brain regions such as the hippocampus and prefrontal cortex (Loubinoux et al., 2012). Recent research has found a significant decrease in brain‐derived neurotrophic factor (BDNF) levels in PSD patients, which affects disease progression and prognosis (Zhang & Liao, 2020). BDNF is a neurotrophic factor involved in the repair and regeneration of damaged neurons. The mitogen‐activated protein kinase (MAPK) signaling pathway plays an important role in the synthesis of BDNF (Figure 1). It is involved in neuronal survival, plasticity regulation, and growth factor signaling. These growth factors include insulin‐like growth factor‐1 (IGF‐1), vascular endothelial growth factor (VEGF), and epidermal growth factor (EGF) (Fernández et al., 2005; Gao et al., 2017). Phosphorylated MAPK3 (also known as extracellular signal‐regulated kinase or ERK1) and its close relative MAPK1 (ERK2) participated in the transcriptional regulation of BDNF through phosphorylation of cAMP response element‐binding protein (P‐CREB). Animal experiments have shown a significant decrease in P‐ERK, P‐CREB, and BDNF levels in the hippocampus of PSD rats (Lim et al., 2014; Zhang et al., 2021).

The gut microbiota is a major microbial community associated with human diseases, influencing host energy metabolism, endocrine metabolism, and immune function. Increasing evidence suggests that altered gut microbiota is related to PSD, attributed to the signaling role of the microbiome–gut–brain axis (MGBA) between the gut microbiota and the central nervous system (Jiang et al., 2021; Mejía‐Granados et al., 2022; Osadchiy et al., 2019). MGBA affects important processes, such as neural transmission, neurogenesis, myelination, and axon formation (Kelly et al., 2021).

Inulin is a natural gut regulator that cannot pass through the intestinal mucosal barrier. Inulin is widely used in food, pharmaceutical, and healthcare industries as a dietary fiber and prebiotic (Gupta et al., 2019). Inulin has multiple confirmed biological functions, including balancing the gut microbiota, correcting endocrine dysfunction, and enhancing immune response (Vandeputte et al., 2017; Wan et al., 2020). Studies have shown that inulin intake can promote the growth of beneficial bacteria in the gut, such as lactobacillus and bifidobacteria (Birkeland et al., 2020; Le Bastard et al., 2020), which are associated with the synthesis of IGF‐1. IGF‐1 is an endocrine hormone that reduces body weight by regulating fatty acid synthesis and lipid metabolism. It can cross the blood–brain barrier and regulate neuronal growth, proliferation, and differentiation through the MAPK signaling pathway (Yang et al., 2021). Rosiglitazone, an insulin sensitizer, exerts its antidepressant effects by promoting the generation of IGF‐1 in the body (Zhao et al., 2017). Therefore, we hypothesize that IGF‐1 is a key signaling molecule in the MGBA, promoting hippocampal BDNF expression through the MAPK/ERK signaling pathway, thereby exerting antidepressant effects. In Alzheimer's disease models, inulin intake has been shown to up‐regulate BDNF expression of hippocampal neurons (Guo et al., 2021). Inulin may be an ideal candidate drug for treating PSD. However, the exact impact and mechanism of inulin, as a prebiotic, on PSD is unknown, and the influence on MGBA needs further clarification.

This study is based on a PSD animal model and aims to investigate whether inulin can regulate the gut microbiota to promote IGF‐1 synthesis, and, through MGBA, modulate the hippocampal MAPK/ERK signaling pathway to enhance BDNF expression, thereby improving depressive behavior in PSD rats. This research may provide a theoretical basis for using inulin as an intervention for central nervous system disorders and offer insights into prevention and treatment strategies for PSD.

## 2 MATERIALS AND METHODS

### 2.1 Acquisition and processing of GEO data

The GSE154434 dataset was downloaded from the NCBI GEO database (Liu et al., 2020). The expression profiles were derived from the hippocampal tissue of mice. To exclude the interference from estrogen on the disease model, four male mice fed with inulin and five male mice with a regular diet were selected as controls. The expression data of the nine samples were normalized using the limma R package. Using the Benjamini–Hochberg method, we calculated the false discovery rate. Differentially expressed genes (DEGs) were obtained by setting the threshold of |log2 Fold Change| > 0.5 and *p*‐value <.05.

### 2.2 Functional annotation of DEGs

The DEGs were annotated using the Kyoto Encyclopedia of Genes and Genomes (KEGG), which assigns specific pathways to high‐level functional information on key data for functional interpretation and practical applications of genomic information (Kanehisa et al., 2017). The Database for Annotation, Visualization, and Integrated Discovery (DAVID) 2021 (https://david.ncifcrf.gov) was used for gene functional analysis. Gene set enrichment analysis (GSEA) was performed to analyze key molecular signaling pathways, and all DEGs involved in the key molecular signaling pathways were used to construct a protein–protein interaction (PPI) network.

### 2.3 Construction of PPI network and selection of HUB genes

The String database (http://string‐db.org) provides evaluation and integration of PPI relationships, including direct (physical) and indirect (functional) associations. String12.0 was used to perform PPI analysis on the target pathway's functional molecules (Miryala et al., 2018). The analysis results were imported into Cytoscape 3.9.1 to build a network model. The top 10 genes with the highest scores according to the Maximal Clique Centrality (MCC) algorithm were used as selection criteria to identify highly interconnected hub genes in the gene expression network (Chaudhary et al., 2019). These genes, as the hub genes with the highest coherence in the signaling pathway network, were evaluated for their importance in various signaling pathways based on the MCC score.

### 2.4 Animal experiment grouping and intervention

A total of 64 male sucrose preference test (SPT)‐grade SD rats were kept in a sterile barrier environment with a temperature of 20–25°C and relative humidity of 45%–65% for 1‐week adaptation (average weight: 170 g, age: 5 weeks). Out of these rats, 56 weighing more than 180 g were selected. After 1 week, we randomly selected six rats using the sample function in R program as the control group. The remaining rats were used to create the middle cerebral artery occlusion (MCAO) model. After a 24‐h post‐surgery period, scores of 1–3, based on the grading criteria proposed by Komine‐Kobayashi et al., were considered successful in establishing the model (Wu et al., 2022). A total of 24 MCAO rats met the scoring criteria and survived after surgery (mortality rate of 23%). To ensure consistent body weight among rat groups before PSD modeling (MCAO + chronic unpredictable mild stress [CUMS]; see next section for more details) and intervention, we sorted the MCAO rats into six groups based on ascending order of body weight. After 7 days post‐surgery, we randomly selected a paper strip with an animal ID from each of the six blind boxes to form new groups (Figure S1). Inulin diet preparation: Add inulin to your daily drinking water at a concentration of 0.3 g/L. According to the instructions for inulin (km445, Chile), the recommended dosage for adults is 0.1–0.15 g/kg, equivalent to 0.04–0.06 g/400 g of rat body weight. Therefore, a 400 g rat with an approximate water intake of 100 mL would consume 0.03 g of inulin for 1 day, which is safe and carries no risk of addiction. The four new groups of MACO rats and one group of normal rats were subjected to the following treatments: (a) Control group (CON), normal rats with a regular diet (without the addition of inulin); (b) PSD group, PSD modeling (MCAO + CUMS), rats with a regular diet; (c) Inulin group (INU), PSD modeling, rats with inulin diet (inulin added to drinking water, concentration: 0.3 g/L); (d) ERK phosphorylation inhibitor group (INU + Inhibitor), PSD modeling, rats treated with an ERK phosphorylation inhibitor (PD98059, HY‐12028, EMC) by intraperitoneal injection (4.8 mL/kg, concentration: 2.08 mg/mL, solvent: dimethyl sulfoxide [DMSO], 33.33 mg/mL, twice a week), with inulin diet; (e) Vehicle group (INU + Vehicle), PSD modeling, rats treated with DMSO by intraperitoneal injection (4.8 mL/kg, concentration: 33.33 mg/mL), twice a week, with inulin diet. This study was approved by the Ethics Committee of the Graduate School of medical, YouJiang Medical University for Nationalities (approval number: 2022052301).

### 2.5 PSD model production

The MCAO model was selected for stroke modeling (Kuts et al., 2019). Anesthesia with 5% isoflurane was administered to the rats after weighing, and then they were fixed. Materials were prepared, and disinfection was performed. A midline incision was made in the neck to separate the external carotid artery, internal carotid artery, and common carotid artery. The external carotid artery and the proximal end of the common carotid artery were ligated. A thread with a round head was inserted from the common carotid artery into the internal carotid artery and slowly advanced to the starting point of the occluded middle cerebral artery, at a depth of 20 ± 2 mm from the bifurcation of the common carotid artery. The internal carotid artery was ligated, and the thread was secured. Excess suture thread was removed after ligation, and the wound was sutured. After surgery, the timer was started, and the animals were placed on a warm blanket (37°C). Anesthesia was reintroduced after 2 h when the animals regained consciousness to remove the thread, resulting in ischemia‐reperfusion. Starting from the seventh day of stroke model establishment, the CUMS protocol based on the Willner method was conducted for 28 days (Hou et al., 2021). Concurrently, the rats received dietary intervention, and their body weight was measured weekly. The groups INU + Vehicle and INU + Inhibitor were given DMSO and inhibitor PD98059, respectively, via intraperitoneal injection. CUMS included the following stressors: 24‐h fasting and water deprivation; 12‐h water deprivation; 12‐h tilted cage (45°); continuous light exposure for 24 h; wet cage (bedding + 200 mL water) for 24 h; 5‐min swimming in 4°C water; 5‐min horizontal shaking; 2‐h behavioral restriction; and 1‐min tail clamp.

### 2.6 Behavioral tests and sucrose preference test

#### 2.6.1 Open field test (OFT)

The rats were placed individually in an unfamiliar open field box (80 cm × 80 cm × 60 cm) and allowed to freely explore for 5 min (Meng et al., 2023). Their spontaneous activity within the past 5 min was recorded using an automated video tracking system (Smart V3.0, Panlab). The locomotor activity of the rats was defined as a measure of depression. Lower activity indicated higher levels of depression. To eliminate the odor left by previous rats, their feces and urine were cleaned with 75% alcohol before each rat underwent the open field test (OFT).

#### 2.6.2 Sucrose preference test (SPT)

On the first day, each group of rats had free access to two bottles of solution containing 1% sucrose solution, and on the second day, they had access to two bottles of solution (one with 1% sucrose solution and the other with water) to adapt to the experiment (Sun et al., 2022). Twenty‐four hours before conducting the SPT, the rats were deprived of food and water as follows: Two bottles, one containing 1% sucrose solution and the other containing water, were provided to the rats at 9 am. The weights of the two bottles were measured after 12 h to collect data for the analysis of the comparison between sucrose solution consumption and water consumption, and the preference was calculated using the formula: preference (%) = sucrose solution intake/total intake × 100. The sucrose preference was monitored in each rat after 28 days of treatment.

### 2.7 Western blot

A RIPA lysis buffer (NCM, No.WB3100) and protease and phosphatase inhibitors were used to lyse rat hippocampal tissue. We measured the protein concentration of the extracts using the BCA protein analysis kit (Beyotime, No.P0010‐1). It was determined that an equal amount of protein (40 mg) was loaded onto the SDS–PAGE gel and then transferred to the PVDF membrane (Immobilon‐P Membrane, IPVH0010). Nonspecific protein binding was blocked with fast blocking solution (Beyotime, No. P0252‐100), followed by incubation with primary antibodies diluted in 1× TBS‐T for 8 h. Primary antibodies used include P‐ERK1/2 (diluted 1:5000, Proteintech, 28733‐1‐AP), ERK1/2 (diluted 1:1000, Proteintech, 11257‐1‐AP), P‐CREB (diluted 1:1000, Affinity, af3189), CREB (diluted 1:500, Abcam, ab32515), BDNF (diluted 1:5000, Abcam, ab108319), and β‐tubulin (diluted 1:5000, Proteintech, 10094‐1‐AP). After primary antibody incubation, the membrane was washed with 1× TBS‐T buffer and then incubated with HRP‐conjugated anti‐rabbit secondary antibody (diluted 1:5000, Proteintech, SA00001‐2) for 1 h. Following another wash, the membrane was visualized using an ECL chemiluminescence kit (NCM, P10100), and images were captured using the Tanon 5200 Multi imaging system (Tanon). Image data analysis was performed using ImageJ software (NIH).

### 2.8 Enzyme‐linked immunosorbent assay

Blood samples were collected in regular tubes and centrifuged at 3500 *g* for 10 min to obtain serum samples. The serum samples were stored at −80°C for subsequent analysis of IGF‐1 levels. IGF‐1 levels in the serum were measured using a commercial assay kit (EIAab, E0050r) following the manufacturer's instructions. The absorbance was read at 450 nm using a spectrophotometer (VICTOR, Nivo), and the serum sample concentrations were calculated using the standard curve equation provided by the kit.

### 2.9 16S DNA gene sequencing of gut microbiota

The analysis of fecal microbiome was conducted using 16S DNA gene sequencing (Fung et al., 2021; Hall & Beiko, 2018). Microbial genomic DNA was extracted using the EZNA Soil DNA Kit (Omega Bio‐Tek). The extracted DNA was checked for quality on a 1% agarose gel and quantified using a UV5Nano UV‐visible spectrophotometer (Mettler Toledo). The V3 and V4 regions of the 16S rDNA gene were amplified through polymerase chain reaction (PCR) using the MiniAmp A37834 thermal cycler (ABI). The PCR amplification protocol for the 16S rDNA gene included initial denaturation at 95°C for 3 min, followed by denaturation at 95°C for 30 s, annealing at 55°C for 30 s, extension at 72°C for 45 s, and a final extension at 72°C for 10 min. The PCR reaction mixture consisted of TransStart FastPfu (5×) buffer, dNTPs, forward and reverse primers, TransStart FastPfu DNA polymerase, template DNA, and double‐distilled water. The PCR reaction was performed in triplicate. The DNA products were obtained from the agarose gel. The extracted DNA was quantified using the Quantus fluorometer (Promega). After library construction was carried out, the library was sequenced using the MiSeq platform.

### 2.10 Statistical analysis

GraphPad Prism v9 (GraphPad Software Inc.) and SPSS 26.0 (IBM Analytics) were used for data analysis. The normality of data was assessed using the Shapiro–Wilk test, whereas the homogeneity of variance was assessed using Levene's test. Data are presented as mean ± standard deviation and analyzed using SPSS software version 26.0 from IBM. Differences between groups were evaluated using one‐way analysis of variance (ANOVA), followed by a post hoc Fisher's least significant difference test. In cases where the data did not follow a normal distribution, the Kruskal–Wallis test was performed, followed by Dunn's test. A significance threshold of *p* < .05 was considered statistically significant. Changes in body weight over time were analyzed using repeated measures ANOVA. To assess the correlation between gut microbiota abundance values and serum IGF‐1 levels, a Spearman correlation analysis was conducted.

## 3 RESULTS

### 3.1 GEO dataset acquisition and differential analysis

**FIGURE 2:**
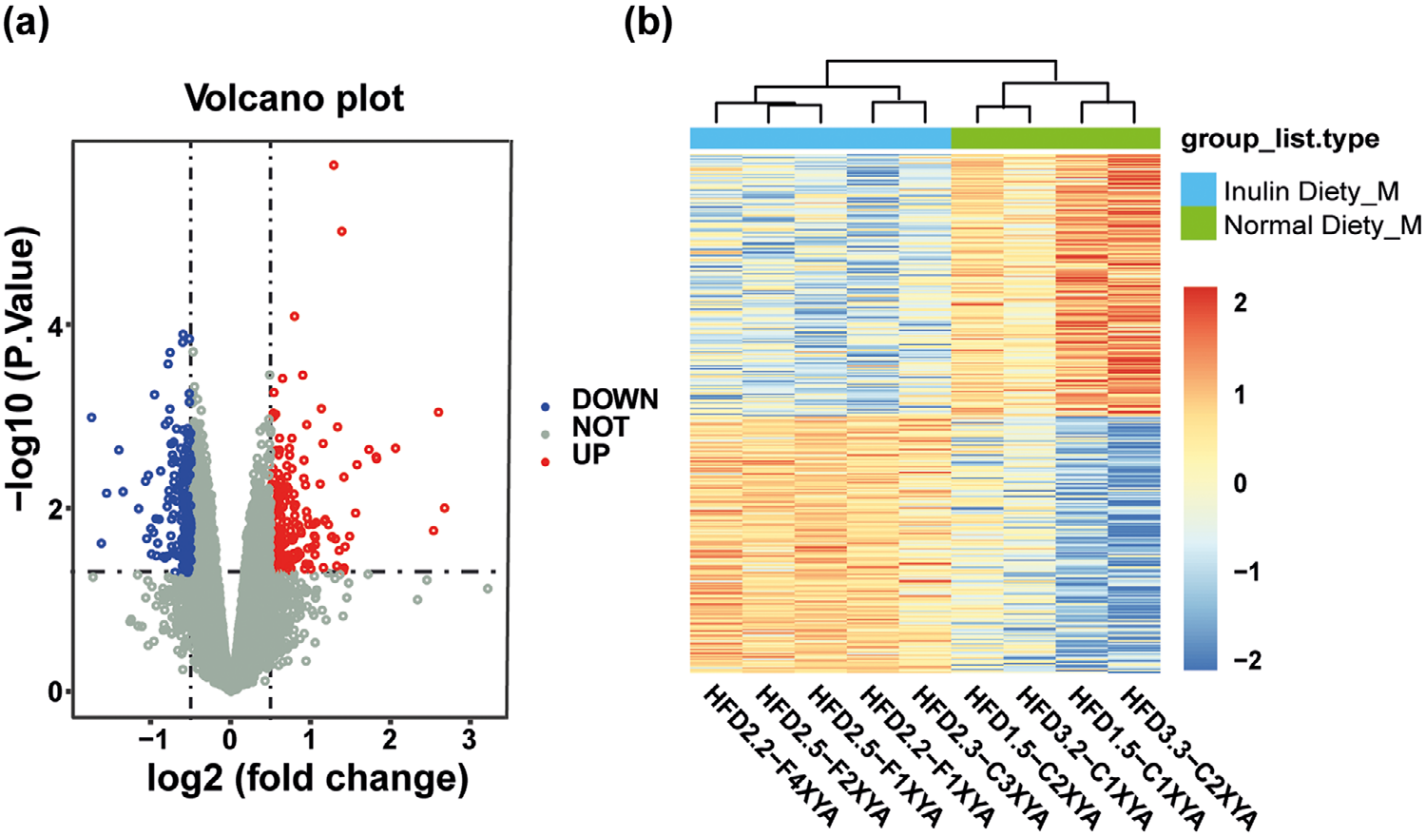
Gene expression profile in the hippocampal tissue of different treatment groups. (a) Red indicates up‐regulated gene expression levels, and blue indicates down‐regulated gene expression levels. (b) Heatmap showing the differential expression changes of DEGs across all samples, with sample clustering displaying intergroup and intragroup differences.

In the GSE154434 dataset, 366 DEGs were identified between the hippocampal tissue samples of the regular diet and inulin intake groups. Among these DEGs, 185 genes were up‐regulated and 181 genes were down‐regulated (Figure 2a). A heatmap was generated to visualize the expression pattern of all the DEGs, which effectively distinguished the samples between the two groups (Figure 2b).

### 3.2 Enrichment analysis of KEGG signaling pathways

**FIGURE 3:**
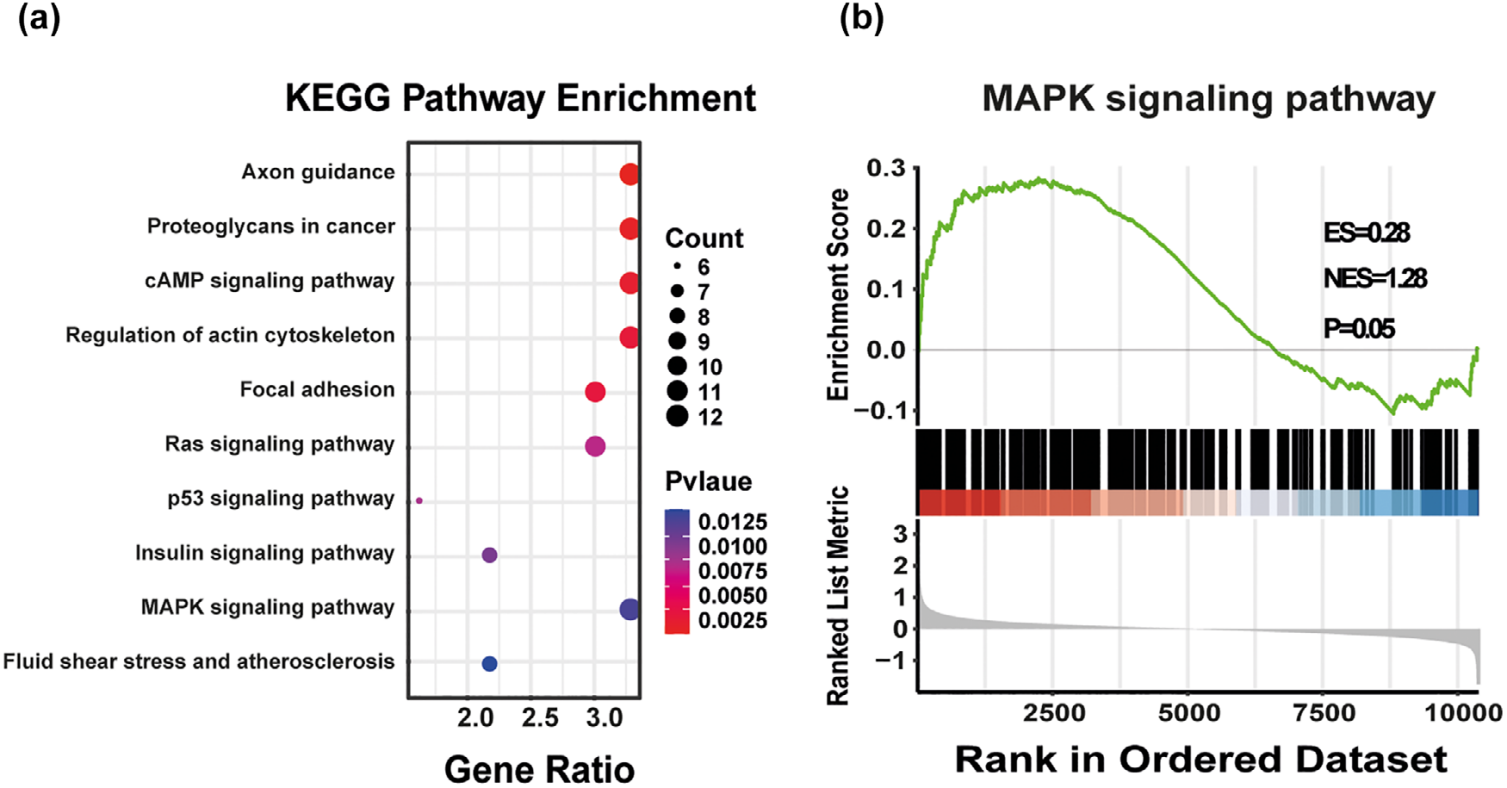
Impact of inulin on Kyoto Encyclopedia of Genes and Genomes (KEGG) signaling pathways. (a) Color represents the significance of differential impact on different pathways. Circle size represents the number of differentially enriched genes in each pathway. (b) Gene set enrichment analysis (GSEA) of the mitogen‐activated protein kinase (MAPK) signaling pathway, with red indicating the proportion of up‐regulated gene expression, and the line represents the enrichment score of the gene (NES: normalized enrichment score; FDR: false discovery rate).

The KEGG functional enrichment analysis revealed the top 10 biological functions associated with the DEGs (Figure 3a). The DEGs were mainly involved in the following 10 pathways. We found that inulin intake significantly affected the MAPK pathway (*p* < .05). Visualization of the MAPK signaling pathway through GSEA showed that the expression levels of most molecules involved in the MAPK pathway were up‐regulated (ES > 0.25, *p* = .05) (Figure 3b).

### 3.3 PPI network of functional molecules in the MAPK pathway

**FIGURE 4:**
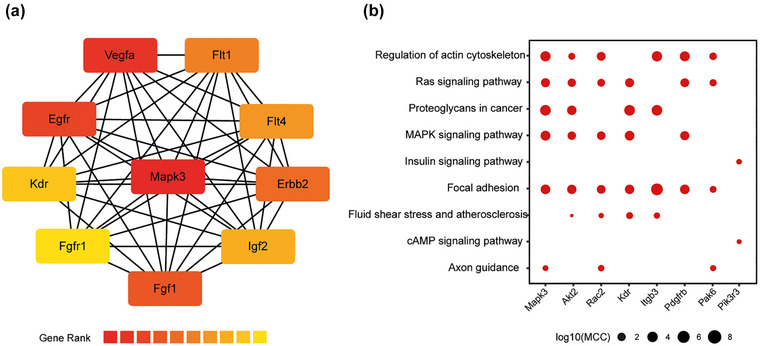
Protein–protein interaction network: (a) central hub gene representing the MAPK signaling pathway; (b) common genes among various signaling pathways, with circle size representing the relative score value of Maximal Clique Centrality (MCC) for each gene in the corresponding pathway.

In the MAPK signaling pathway, MAPK3 (ERK1) was identified as the hub gene with the highest degree of connectivity (Figure 4a). MAPK3 acts as a crucial hub gene and regulates six key signaling pathways (Figure 4b).

### 3.4 Inulin regulated the expression levels of various molecules in the hippocampal MAPK signaling pathway

**FIGURE 5:**
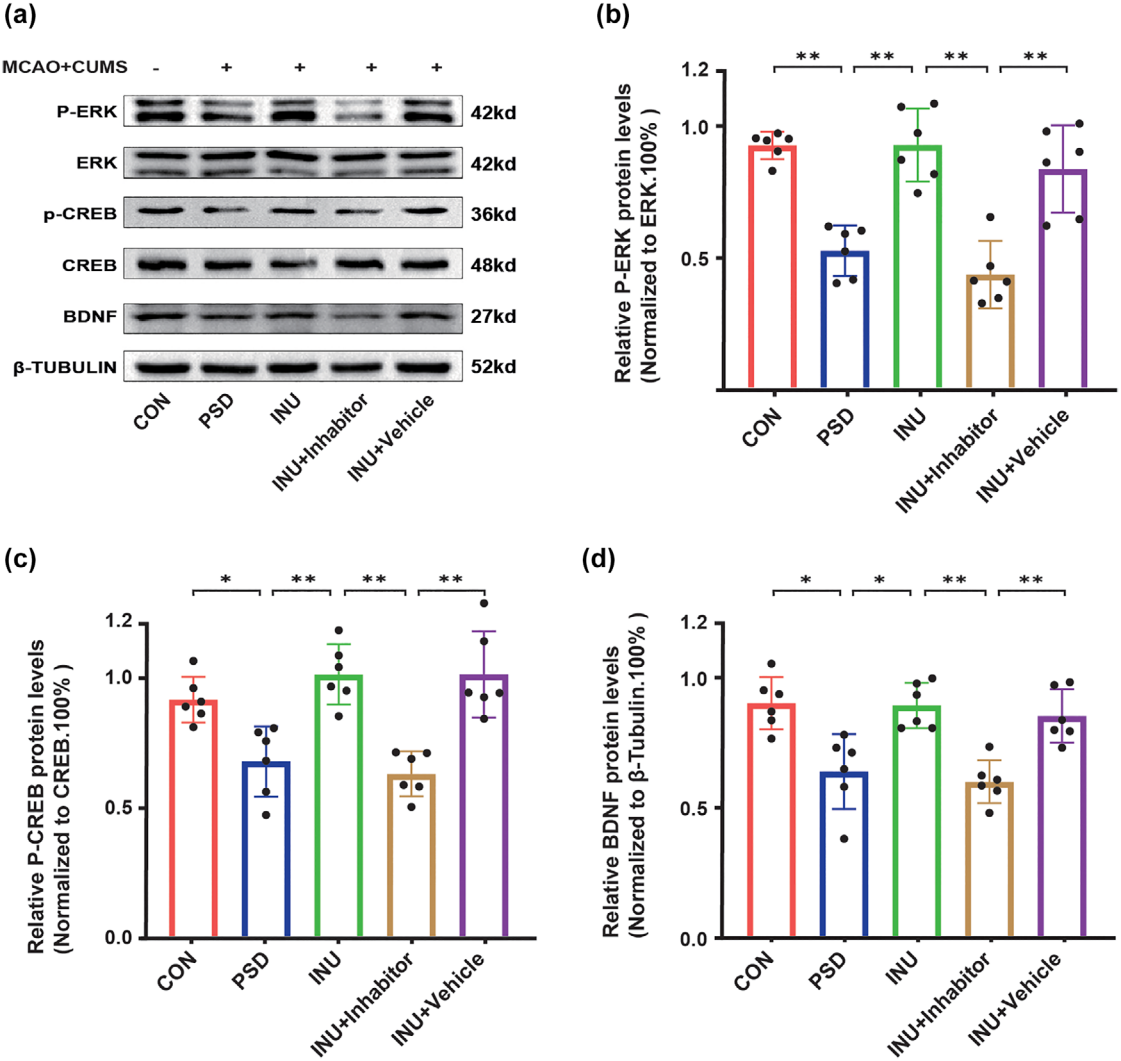
Western blot analysis of protein expression levels: (a) This figure shows the protein expression levels of each group, sourced from rat hippocampal tissue; (b) the ratio of P‐ERK to ERK in different groups; (c) the ratio of P‐CREB to CREB in each group; (d) changes in brain‐derived neurotrophic factor (BDNF) expression levels in different groups. *p < .05, **p < .01.

Hippocampal neurons play an important role in cognition and emotional control, and apoptosis of neuronal cells can lead to mental disorders (Liu et al., 2017). Furthermore, recent studies have directly confirmed that neuronal reduction can cause anxiety and depression with neuro‐pathological consequences, whereas high expression of BDNF promotes neuronal regeneration and repair, thereby alleviating depressive behavior (Gold, 2021). Our experiment also indicates that the phosphorylation levels of ERK and CREB in the PSD rat hippocampal area are significantly decreased compared to CON, and the levels of P‐ERK and P‐CREB is significantly increased in INU compared to PSD. In addition, the expression level of BDNF is increased in INU. When comparing INU + Inhibitor with INU, the use of the inhibitor significantly inhibits the phosphorylation of ERK, leading to a significant decrease in the levels of P‐CREB and BDNF. The use of the Vehicle does not affect the protein levels of the various functional molecules (Figure 5).

### 3.5 Inulin dietary alters the diversity and abundance of the gut microbiota in PSD rats

**FIGURE 6:**
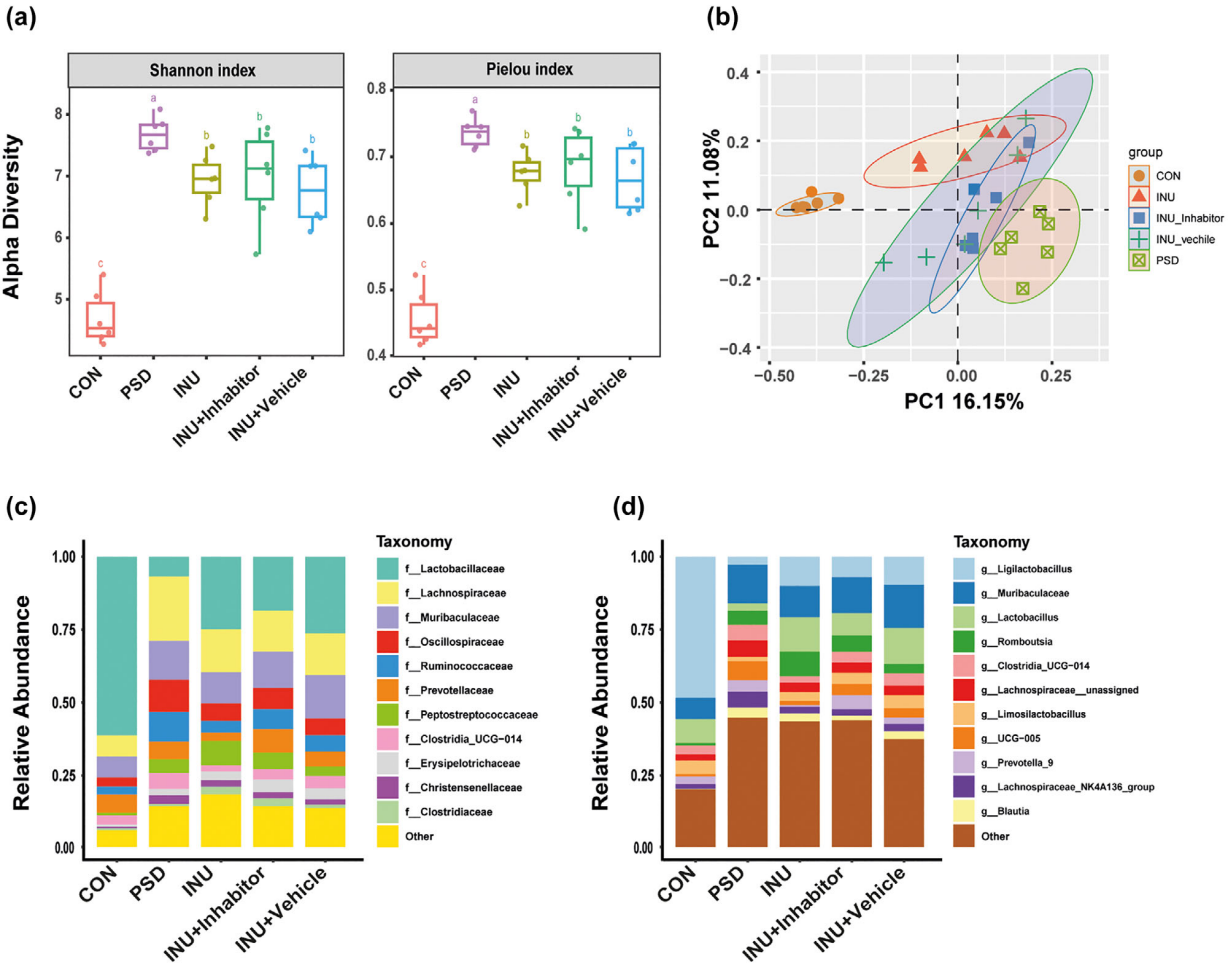
Species diversity and abundance. (a) Microbial alpha diversity (calculated based on original abundance, *n* = 6): represented by Shannon index and Pielou index. Groups labeled with the same letter indicate no statistical differences. (b) Microbial beta diversity: Each point represents a sample, and closer distances between points indicate greater similarity. The *x*‐ and *y*‐axes represent the contribution rates of the first and second principal components, respectively. (c) Relative abundance of species at the family level. (d) Relative abundance of species at the genus level.

Compared to other groups, PSD rats exhibited increased species alpha diversity of their gut microbiota, primarily reflected on the Shannon and Pielou indices. However, after inulin intake, the species diversity of the PSD rats significantly decreased. There were no statistically significant differences in the alpha diversity of INU, INU + Inhibitor, and INU + Vehicle (Figure 6a). The beta diversity of the gut microbiota showed that CON, INU, and PSD groups were well separated in terms of spatial distance, as indicated by the first and second principal components (Figure 6b).

At the family level, the top five abundant families were Lactobacillaceae, Lachnospiraceae, Muribaculaceae, Oscillospiraceae, and Ruminococcaceae. Lactobacillaceae accounted for 60% in CON. After PSD induction, the abundance of Lactobacillaceae decreased by more than 40%, whereas other species showed an increase. Inulin intake increased the abundance of Lactobacillaceae and reduced the abundance of Lachnospiraceae, Oscillospiraceae, and Muribaculaceae compared to PSD group. INU + Inhibitor, INU + Vehicle, and INU displayed similar species abundances (Figure 6c).

At the genus level, compared to CON, the abundance of *Ligilactobacillus*, *Lactobacillus*, and *Limosilactobacillus* decreased by more than 50% in PSD group, except for Prevotella_9. However, inulin intake reversed this change. Unlike the gut microbiota in CON, the abundance of inflammatory microorganisms increased in the gut of PSD rats, including Lachnospiraceae_NK4A136_group, Oscillospiraceae_UCG_005, Muribaculaceae, *Romboutsia*, *Ruminococcus*, *Blautia*, and Clostridia_UCG_014. Except for Romboutsia and Muribaculaceae, inulin supplementation also reduced the abundance of these species. There is no statistically significant difference among INU, INU + Inhibitor, and INU + Vehicle, except for Muribaculaceae and Prevotella_9 (Figure 6d).

### 3.6 The influence of inulin diet on the structural characteristics of the gut microbiota in PSD rats

**FIGURE 7:**
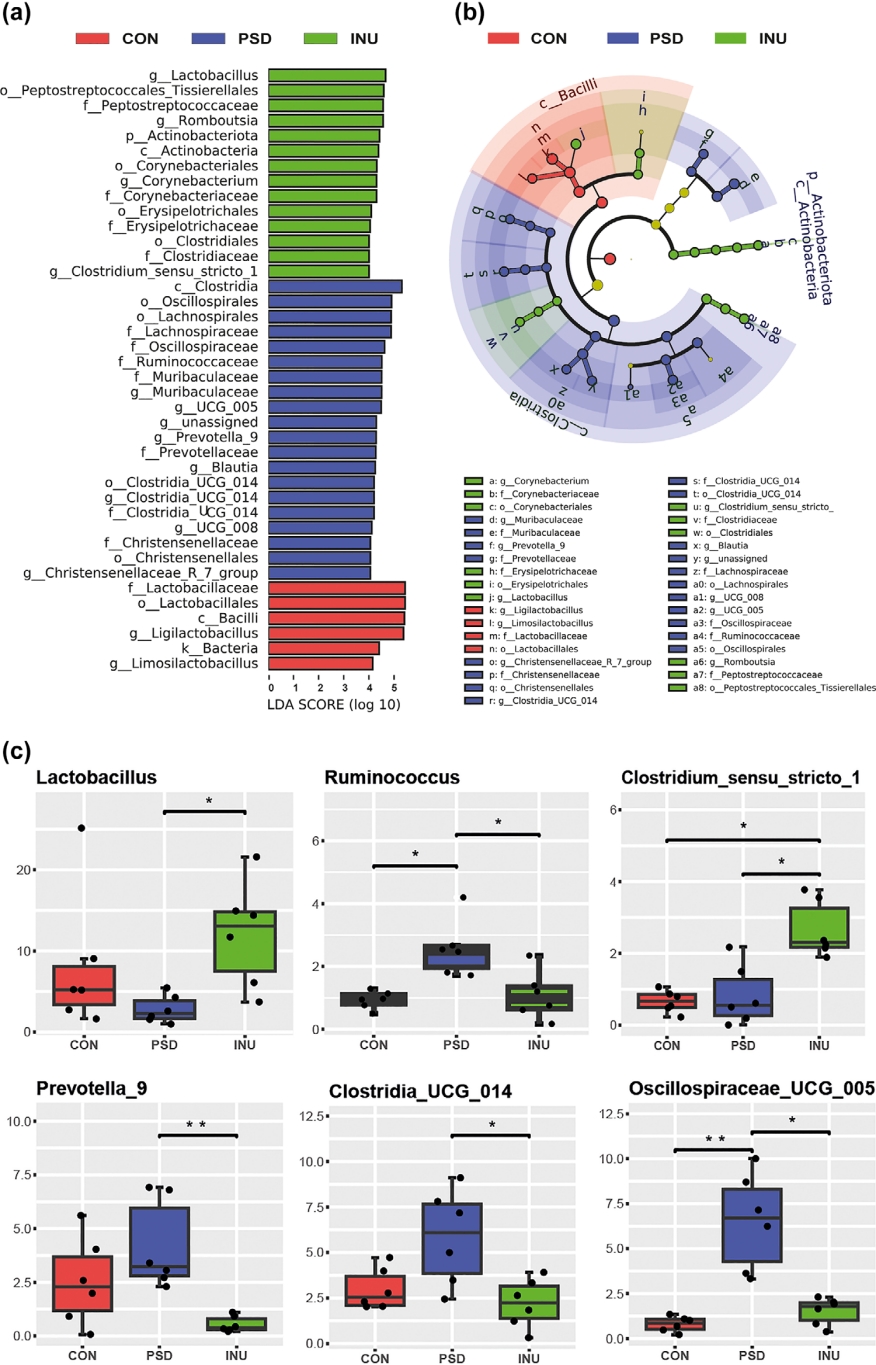
Linear discriminant analysis effect size (LEFSe) and relative abundance analysis of species. Part (a) shows the significantly different species abundances among different groups, where the length of the bars represents the magnitude of the impact of the different species. (b) The nodes of different colors to represent significantly enriched microbial taxa in the corresponding populations, with the radiating branches representing classification levels from kingdom to genus, and the size of the circles representing the relative abundance. The differentially significant species biomarkers are color‐coded based on the groups they follow. (c) Box plots of inter‐group differences at the genus level. Inter‐group analysis of differences in relative abundance of gut microbiota at the genus level. **p < .01, *p < .05.

The previous analysis indicated that the use of ERK phosphorylation inhibitors and vehicle did not significantly alter microbial diversity and species abundance. In this study, we performed LEFSe analysis on CON, INU, and PSD groups to identify statistically different biological markers among groups. The LDA score was used to evaluate the magnitude of the impact of different species. Bacilli, Lactobacillales, Lactobacillaceae, and Ligilactobacillus were significantly enriched in CON (LDA score >5), whereas Clostridia, Clostridiaceae, Lachnospiraceae, Oscillospiraceae, and Muribaculaceae were predominant in the gut microbiota of PSD rats. In INU, the enrichment of *Lactobacillus* was the most significant (Figure 7a). The LEFSe cladogram (Figure 7b) revealed that characteristic gut microbiota in INU and CON both originated from the phylum *Firmicutes* and order Lactobacillales. After INU intake, in addition to *Lactobacillus*, the characteristic gut microbiota also included Actinobacteriota, Actinobacteria, Bifidobacteria, Clostridia, and Clostridiaceae, which could be distinguished from CON and PSD groups. Several characteristic microbiotas with biological markers in INU and PSD groups were derived from the order Clostridia.

At the genus level of gut microbiota, a significant difference in microbial diversity is observed with the consumption of inulin diet. Here, we present the six genera of gut microbiota that are most significantly affected by inulin intake compared to the PSD group (Figure 7c), including *Lactobacillus*, *Ruminococcus*, Clostridium_sensu_stricto_1, Prevotella_9, Clostridia_UCG_014, and Oscillospiraceae_UCG_005. We observed that, compared to CON, the relative abundance of *Ruminococcus* and Oscillospiraceae_UCG_005 in the gut microbiota of PSD rats was significantly increased. However, after inulin intake, both showed a significant decrease. Furthermore, compared to PSD rats, we found that inulin intake increased the relative abundance of *Lactobacillus* and Clostridium_sensu_stricto_1 in the gut. Additionally, apart from reducing the relative abundance of *Ruminococcus* and Oscillospiraceae_UCG_005, the inulin diet also decreased the relative abundance of Prevotella‐9 and Clostridia_UCG_014.

### 3.7 The plasma levels of IGF‐1 are associated with the gut microbiota

**FIGURE 8:**
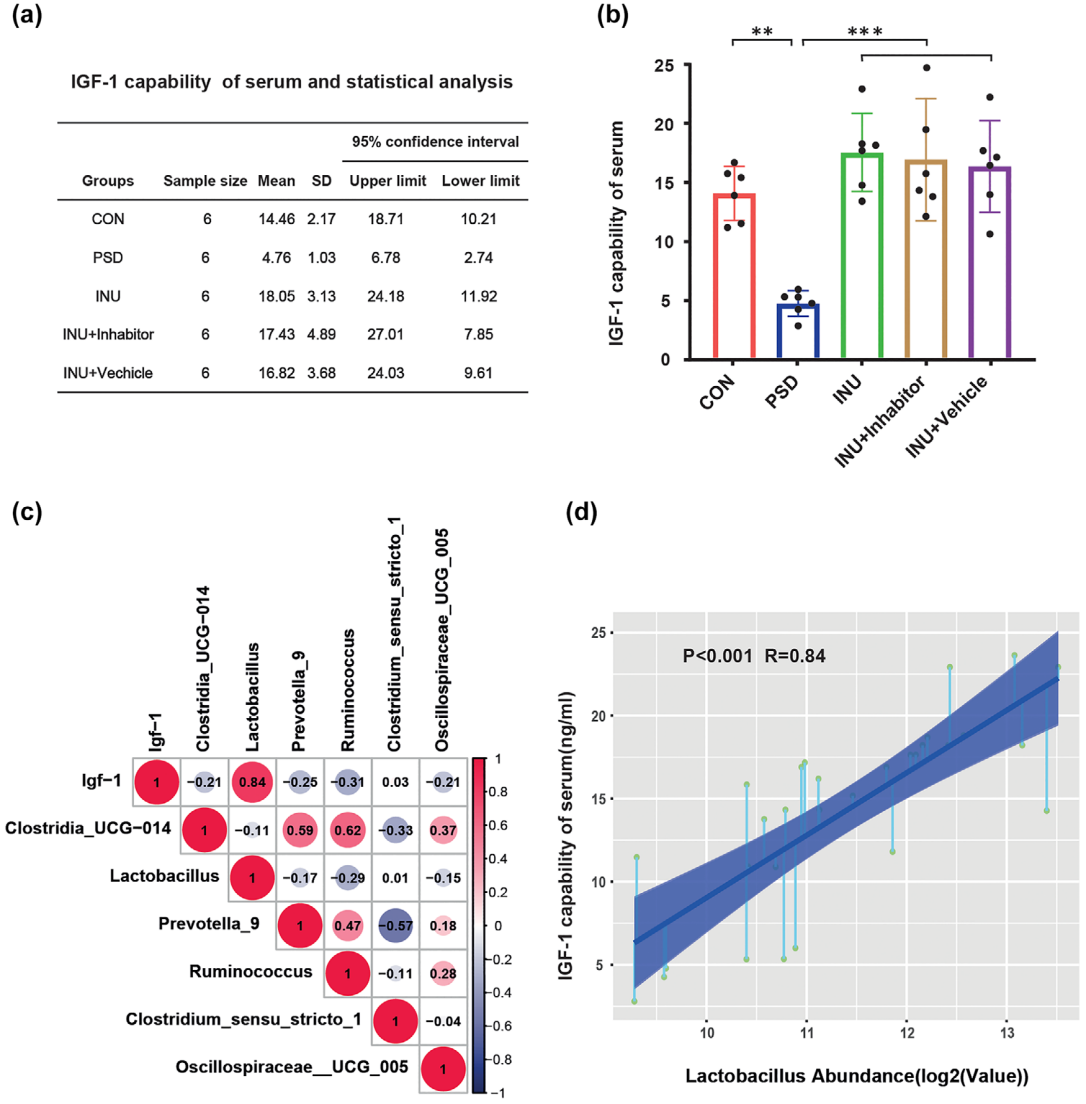
The levels of insulin‐like growth factor 1 (IGF‐1) of serum and correlation analysis: Table (a) displays the quantitative levels of IGF‐1 in serum (unit: ng/mL); (b) analysis of the differences in IGF‐1 values in the serum of each group of rats; (c) correlation analysis between differential microbes and serum IGF‐1 levels, where the size of the circles represents the correlation coefficient values. Red indicates positive correlation, whereas blue indicates negative correlation; (d) correlation analysis between *Lactobacillus* and serum IGF‐1 levels.

Our measurement results show that the normal rat serum IGF‐1 level is 14.46 ± 2.7 ng/mL (Table A in Figure 8). The serum IGF‐1 level in PSD rats was significantly reduced, whereas it significantly increased after ingestion of inulin. There was no difference in serum IGF‐1 levels among INU, INU + Inhibitor, and INU + Vehicle (Figure 8b). The levels of IGF‐1 have a strong correlation with *Lactobacillus*, whereas they have a negative correlation to some extent with Ruminococcus (Figure 8c,d).

### 3.8 Inulin diet alleviated depressive‐like behavior and reduced weight in PSD rats

**FIGURE 9:**
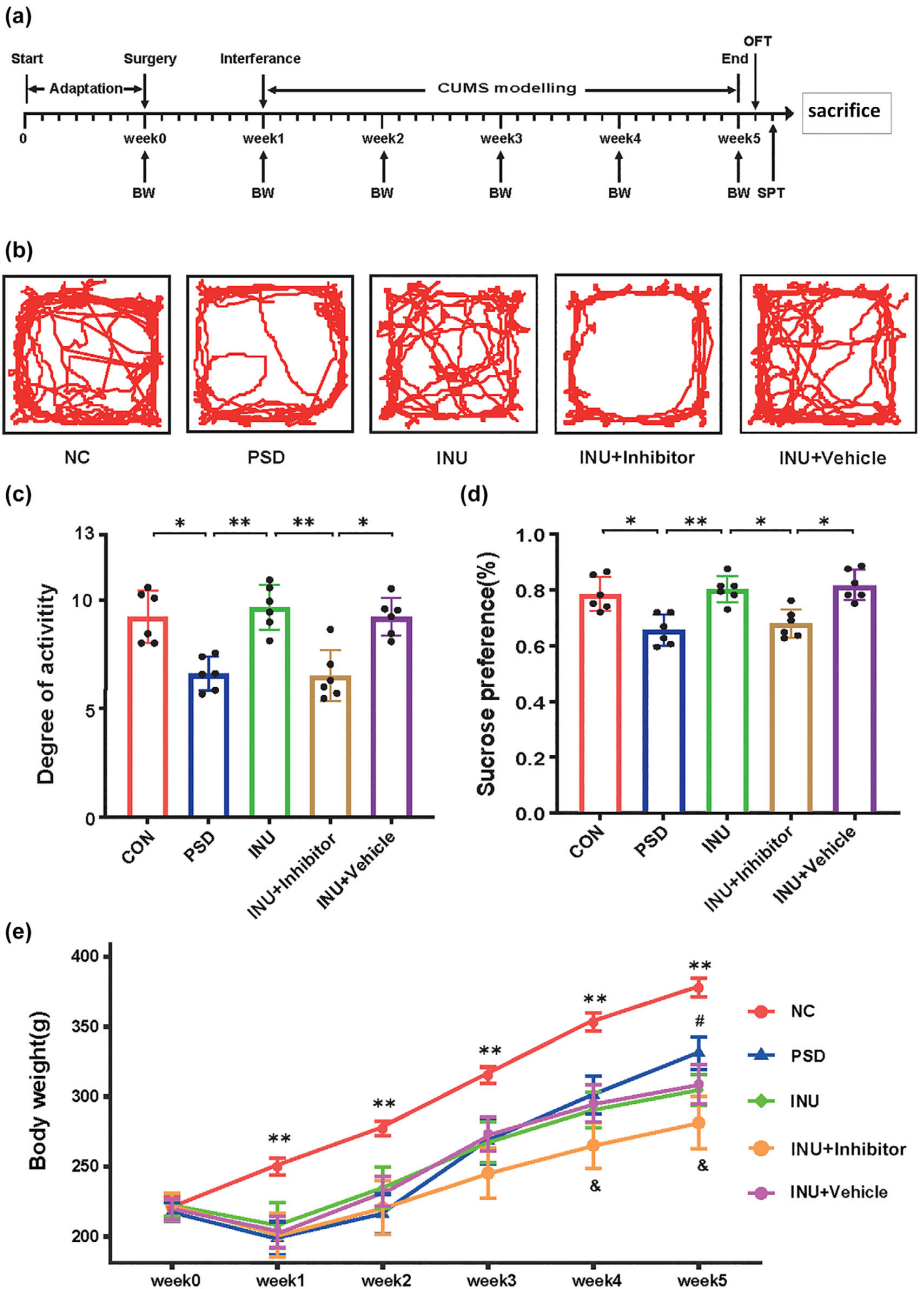
Animal behavior and weight changes. (a) Timepoint table for the experimental procedure. CUMS, chronic unpredictable mild stress; BW, rat weight measurement; OFT, open field test; SPT, sucrose preference test. Part (b) shows the activity trajectory of rats. Part (c) depicts OFT with the *x*‐axis representing groups and the *y*‐axis representing rat activity. Part (d) represents the sucrose preference test, with the *y*‐axis indicating the sugar consumption rate of rats and the *x*‐axis representing groups. The data are presented as mean ± SD (*n* = 6). *p < .05, **p < .01. (e) Line graph showing the weight change of rats. “**” represents the comparison between CON and PSD groups, *p* < .05. “#” represents the comparison between INU and PSD groups, *p* < .05. “&” represents the comparison between INU + Inhibitor and INU groups, indicating *p* < .05.

The behavior test and body weight of rats were monitored according to the time points of the experimental procedure (Figure 9a). The effects of inulin on depressive‐like behavior in PSD rats were evaluated using the OFT and SPT. The movement trajectory of the rats was used to reflect their interest in exploring unfamiliar environments (Figure 9b). We observed a significant decrease in the number of center zone activities in the rats. The activity index in the OFT was used to assess the motor function of the rats (Figure 9c). The activity index of both PSD and PSD + Inhibitor decreased significantly. The activity level of the rats in the PSD group was significantly lower than that in CON. Compared to the PSD group, the activity level of the rats in INU increased significantly. When comparing INU with INU + Inhibitor, there was a significant decrease in the activity index of rats after the use of the inhibitor. The use of the Vehicle did not have a significant effect on rat behavior. The sucrose preference in the SPT was used to reflect the desire for pleasant stimuli in the rats (Figure 9d). The analysis results showed that the sugar preference rate in the PSD group was significantly lower than that in CON. INU significantly increased the sucrose preference in PSD rats. INU + Inhibitor showed a significant decrease in sugar water preference compared to PSD, with statistically significant differences. The body weight displayed through a line graph showing the changes in body weight (Figure 9e). Compared to CON, the body weight of PSD rats began to decrease after 1 week. After 1 month of inulin intake, the body weight of the INU and INU + Inhibitor further decreased compared to the PSD group. In comparison to INU, INU + Inhibitor also showed a decrease in the fourth and fifth weeks, with statistically significant differences.

## 4 DISCUSSION

This study emphasizes the importance of gut microbiota in the pathophysiology of PSD and aims to improve the depressive phenotype of PSD by balancing the gut microbiota. The study found a decrease in beneficial bacteria (*Lactobacillus*) and an increase in harmful bacteria (*Ruminococcus*, Oscillospiraceae) after PSD. This is consistent with the findings of Cai et al. (2023), further confirming the reliability of our results. The gut–brain axis plays an important role in bidirectional communication between the gut microbiota and the central nervous system, with critical functions in substance metabolism, neurotransmitters, and neuroinflammation regulation. Brain ischemic injury caused by stroke leads to systemic inflammatory responses and the production of various inflammatory mediators such as TNF‐α, IL‐6, and IL‐1β (Daidone et al., 2021; Tuttolomondo et al., 2016), which are also involved in the neuroinflammatory mechanisms of PSD (Wei et al., 2021). These inflammatory mediators disrupt gut barrier function and microbial balance, further exacerbating the inflammatory state, impacting stroke recovery, and promoting the development of PSD. In addition, PSD is also influenced by both brain ischemic injury and chronic stress, which interfere with normal gut function, leading to digestive system disorders including changes in intestinal motility, damaged mucosal barriers, and dysbiosis (Jiang et al., 2023). Gut microbiota produces various signaling molecules and metabolites, such as neurotransmitters, hormones, and short‐chain fatty acids (SCFAs) (Panther et al., 2022). When these substances undergo changes, they can cross the blood–brain barrier through the peripheral circulation and impact brain regions related to mood and cognitive function, ultimately promoting the occurrence of PSD. *Ruminococcus* and Oscillospiraceae are strict anaerobic bacteria in the gut that produce inflammatory polysaccharides (Henke et al., 2019). Studies have shown that inflammatory polysaccharides can activate the NFKB signaling pathways through the blood–brain barrier to participate in the activation of neuroinflammation (Savran et al., 2020). Although there is no direct experimental evidence linking increased *Ruminococcus* and Oscillospiraceae in the gut to an increased risk of PSD, a recent study observed that an increase in gut *Ruminococcus* in a depression rat model leads to an increased risk of depression (Lukic et al., 2019). This indirectly provides evidence for the involvement of gut microbiota in the occurrence and development of PSD.

Of course, the gut microbiota not only participates in the inflammatory mechanisms of PSD but also plays a role in regulating SCFAs. It has been reported that *Lactobacillus*, *Ruminococcus*, and *Bacteroides* can enhance the synthesis of SCFAs in the gut. However, our research contradicts this as we found that inulin intake reduced the abundance of *Ruminococcus* and *Bacteroides* but significantly increased the relative abundance of *Lactobacillus*. Overall, the proportion of microbiota producing SCFAs was significantly higher compared to the control group. SCFAs produced by microbial metabolites can maintain the levels of IGF‐1 in serum and mesenteric lymph nodes and promote the production of IGF‐1 through liver metabolism (Matsushita et al., 2022; Yuan et al., 2021). Our experiments did not confirm whether the increase in IGF‐1 after inulin intake is related to the synthesis of SCFAs. Further measurements of SCFAs in feces and serum are needed for clarification. However, our study at least indicates that inulin intake can increase the level of IGF‐1 hormone in the body. Zhang et al. (2018) found that all PSD patients had low serum levels of IGF‐1 in a study of 32 clinical samples. We confirmed that after the appearance of depressive phenotype in PSD rats, the level of IGF‐1 in their serum decreased significantly, which suggests that these changes may be related to the pathophysiology of depression symptoms in stroke patients. The increase in IGF‐1 levels due to inulin may indirectly provide new intervention targets for preventing depressive symptoms in stroke patients. Our study found a noticeable downward trend in body weight in PSD rats after 3 weeks of inulin diet intervention. This conclusion is based on the increase in *Lactobacillus* abundance with inulin supplementation and the positive correlation between *Lactobacillus* abundance and IGF‐1 levels confirmed by correlation analysis. The impact on body weight can be attributed to the role of IGF‐1 in improving fat distribution and weight control through increased fat oxidation, inhibition of fat synthesis, and promotion of skeletal muscle growth (Kunitomi et al., 2002; Takahashi, 2017). This aligns with the effect of using *Lactobacillus* to improve obesity in obesity models (Chen et al., 2022).

In our study, we have discovered that feeding mice with inulin has an up‐regulating effect on the MAPK signaling pathway in the hippocampus. This finding was derived from the analysis of transcriptomic data from mouse hippocampus. Previous studies have consistently shown a down‐regulation of the MAPK signaling pathway in the hippocampus of both mice and rats with PSD (Qinlin et al., 2022; Zhang et al., 2021). Additionally, the study conducted by Feng et al. (2022) further supports the notion that pyruvate kinase M2 improves post‐stroke depressive symptoms by activating the VEGF‐mediated MAPK/ERK pathway. Furthermore, a notable study has demonstrated a down‐regulation of the ERK–CREB–BDNF signaling pathway in mice models of chronic stress‐induced depression (Cai et al., 2023). These research findings provide further support and strengthen the feasibility of our study.

In all our PSD models, rats that consumed inulin for one month showed weight loss compared to rats on a normal diet, with the most significant reduction observed in rats treated with an ERK phosphorylation inhibitor. The ERK signaling pathway also plays a key role in the development and metabolism of adipocytes. Inhibiting the ERK signaling pathway can reduce the proliferation and differentiation of fat cells, thereby reducing the formation and accumulation of adipose tissue (Wu et al., 2022). The use of an ERK phosphorylation inhibitor disrupts the expression of genes related to fat synthesis, thereby affecting the development and function of fat cells. Additionally, the ERK signaling pathway in certain regions of the brain, such as the hypothalamus and pituitary gland, is involved in regulating appetite and energy metabolism (Kwon et al., 2016).

The MAPK signaling pathway promotes the growth and differentiation of neurons, which is regulated by multiple growth factor signals. Our study only confirmed that increased levels of IGF‐1 in the circulatory system after inulin intake promoted the activation of the MAPK pathway, but it does not prove whether other growth factors also contribute to the activation of the MAPK pathway. Further determination of the levels of other growth factors such as VEGF and EGF is needed for supporting evidence. However, it was indeed observed that the phosphorylation levels of ERK and CREB increased after inulin intake. An early study found that inhibiting ERK phosphorylation can produce antidepressant effects by injecting BDNF into the hippocampus (Shirayama et al., 2002). Pre‐treatment with PD98059 inhibited the activation of P‐ERK, P‐CREB, and the expression of BDNF in the hippocampus of rats after inulin intake. The hippocampus plays an important role in cognition and emotional control. Long‐term PSD can cause apoptosis of hippocampal neurons and increased autophagy, leading to a decrease in the number of neurons (Sun et al., 2020; Wang et al., 2021). These studies indicate that the reduction of neurogenesis in the hippocampus is related to the pathogenesis of anxiety and depression, and BDNF is involved in neuroprotection and synaptic plasticity in the central nervous system.

Some limitations of our study include the following: (1) Although our findings suggest an association between dysbiosis of the gut microbiota and PSD, conducting fecal transplantation experiments would strengthen our ability to establish causal relationships. (2) Our study was limited to male rats, and it is important to investigate whether similar results are observed in female rats. Further experimentation and validation in female subjects are warranted. (3) The use of 16S DNA sequencing technology restricted our ability to identify bacterial strains at the species level, limiting our understanding of specific microbial contributions. Future studies employing shotgun metagenomic sequencing methods could provide more detailed strain‐level information. (4) Given the known involvement of gut microbiota in PSD development and its interaction with MAPK signaling pathways, further investigation is necessary to identify specific key regulatory strains or microbial factors involved in these processes.

In summary, through microbiome data and transcriptome data, we have outlined the changes in the microbiota of PSD rats. We have concluded that the gut microbiota may be involved in the development of PSD, with mechanisms related to the MAPK signaling pathway. However, more mechanisms of the microbiota–brain–gut axis need to be further explored. The results of this study provide a new perspective for understanding the prevention and treatment of PSD.

## AUTHOR CONTRIBUTIONS

**Rong Shao**: Software; methodology; conceptualization; writing—original draft; supervision; validation. **Xiongchang Tan**: Formal analysis; resources; investigation; data curation; validation. **Minfu Pan**: Visualization; resources. **Jiawen Huang**: Resources; visualization; investigation; formal analysis. **Liu Huang**: Visualization; resources. **Binyu Bi**: Conceptualization; methodology. **Xiaohua Huang**: Visualization; resources. **Jie Wang**: Project administration; funding acquisition; supervision; writing—review and editing. **Xuebin Li**: Writing—review and editing; funding acquisition; supervision; project administration.

## CONFLICT OF INTEREST STATEMENT

The authors declare no potential conflicts of interest with respect to the research, authorship, and/or publication of this article.

### PEER REVIEW

The peer review history for this article is available at https://publons.com/publon/10.1002/brb3.3387.

## Supporting information

Supporting Information

## Data Availability

The datasets used and/or analyzed during the current study are available from the corresponding author on reasonable request.
