# Supplementary figures and images for: Inulin alters gut microbiota to alleviate post‐stroke depressive‐like behavior associated with the IGF‐1‐mediated MAPK signaling pathway
Source: Brain Behav. 2024 Jan 17;14(1):e3387. doi: 10.1002/brb3.3387 (PMC10794126; doi:10.1002/brb3.3387)

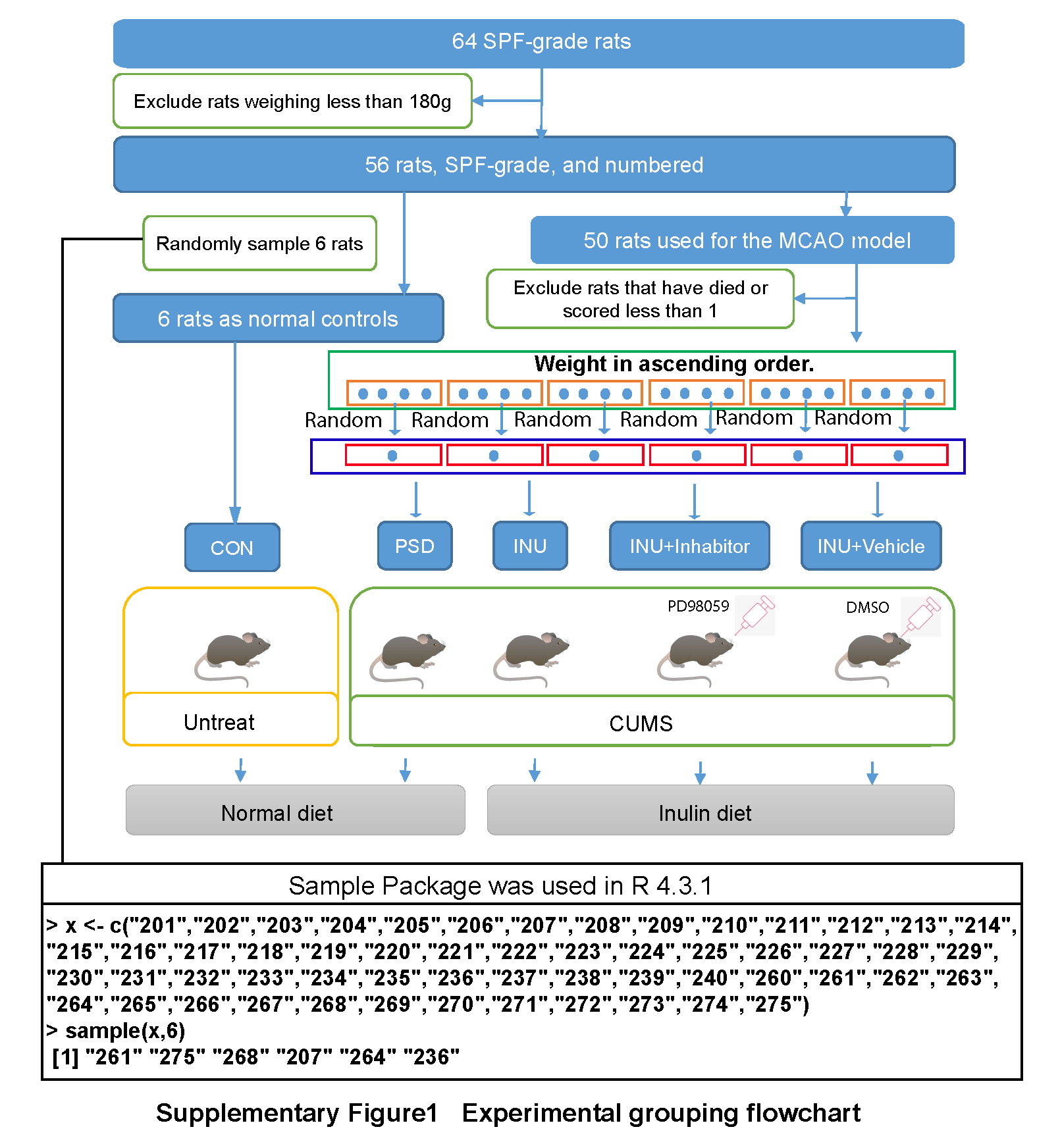

Supplement: Supplementary file 1 — Supporting Information [file BRB3-14-e3387-s001.tif]
